# Designing function-specific minimal microbiomes from large microbial communities

**DOI:** 10.1038/s41540-024-00373-1

**Published:** 2024-05-03

**Authors:** Aswathy K. Raghu, Indumathi Palanikumar, Karthik Raman

**Affiliations:** 1 Centre for Integrative Biology and Systems mEdicine (IBSE), Indian Institute of Technology (IIT) Madras, Chennai, 600 036 India; 2 Robert Bosch Centre for Data Science and Artificial Intelligence (RBCDSAI), IIT Madras, Chennai, 600 036 India; 3 Department of Biotechnology, Bhupat Jyoti Mehta School of Biosciences, IIT Madras, Chennai, 600 036 India; 4 Department of Data Science and AI, Wadhwani School of Data Science and AI, IIT Madras, Chennai, 600 036 India; 5 Present Address: Department of Chemical and Biological Engineering, Northwestern University, IL, 60208 USA

**Keywords:** Systems biology, Software

## Abstract

Microorganisms exist in large communities of diverse species, exhibiting various functionalities. The mammalian gut microbiome, for instance, has the functionality of digesting dietary fibre and producing different short-chain fatty acids. Not all microbes present in a community contribute to a given functionality; it is possible to find a *minimal* microbiome, which is a subset of the large microbiome, that is capable of performing the functionality while maintaining other community properties such as growth rate and metabolite production. Such a minimal microbiome will also contain keystone species for SCFA production in that community. In this work, we present a systematic constraint-based approach to identify a minimal microbiome from a large community for a user-proposed function. We employ a top-down approach with sequential deletion followed by solving a mixed-integer linear programming problem with the objective of minimising the *L*_1_-norm of the membership vector. Notably, we consider quantitative measures of community growth rate and metabolite production rates. We demonstrate the utility of our algorithm by identifying the minimal microbiomes corresponding to three model communities of the gut, and discuss their validity based on the presence of the keystone species in the community. Our approach is generic, flexible and finds application in studying a variety of microbial communities. The algorithm is available from https://github.com/RamanLab/minMicrobiome.

## Introduction

Microorganisms seldom exist in isolation in nature; they form communities and survive by interacting with other microbial species. Communities are known to exhibit cooperation and competition depending on the composition of member species and environmental conditions^1^. The human gut harbours at least 1000 different species, comprising 10^13^–10^14^ organisms, whose collective genome is at least 100 times the human genome^2^. The gut microbiome is an essential part of the digestive system that synthesizes amino acids and vitamins and breaks down the otherwise indigestible dietary fibre into products that the human body can absorb^3,4^. It has a massive influence on human health, and its disruption has been found to cause several disease states^5^ such as obesity^6,7^, type II diabetes^8^ and inflammatory bowel disease^6^. Microbes in the gut are known to co-exist by cross-feeding metabolites^9^ and by performing complementary metabolic functions^1^. Gut microbes predominantly survive on the dietary fibre, glycans and secretions from the host epithelial cells^10^. The symbiotic association between humans and the gut microbiome is demonstrated through the role of microbiome-derived short-chain fatty acids (SCFA), bile acids and other small molecules in maintaining energy homeostasis and regulating gut barrier and inflammation^11^. SCFA are involved in adaptive immune system response, provide energy for the growth of colon epithelial cells, play a role in cholesterol synthesis, and are involved in the crosstalk with other tissues like the lung and liver^3,12,13^. For instance, butyrate has been reported to have a significant influence on maintaining host health owing to its ability to induce apoptosis^14^, develop intestinal barrier^15^ and regulate the immune system^16^. Perturbation to the community, such as that brought about by antibiotic usage^17–19^, can disturb the community and affect its functionality. All the members of the microbial community do not contribute equally to the production of all the metabolites. Only some of them have the capability to break down dietary fibre, and such species are regarded as keystone species in the community^20,21^. For example, certain species of *Bifidobacterium*, *Bacteroides* and *Firmicutes* can degrade polysaccharides^22^. The numerical abundance of such organisms may not be significant compared to their functional impact on the community^20,21^. Microbiomes also possess functional redundancy due to which dissimilar organisms capable of similar functions can be interchanged^21^. Understanding the interaction characteristics of the member species is paramount to comprehend the features of the overall microbiome, and several works have been published in this regard^20,23^. Previous literature has shown that synthetic microbial communities comprising representative species from major phyla present in the gut^24^ can be used for treating diseases such as *Clostridium difficile* infection (CDI) and Inflammatory Bowel Disease (IBD)^25^.

Computational tools are very effective and widely used for studying the behaviour of microbes in a community using their genome-scale metabolic models. Numerous approaches using genome-scale metabolic networks (GSMNs) at varying levels of complexities are available for microbial community modelling^26–31^. Algorithms such as MiMiC^32^ use data-driven approaches on microbial metagenomes to predict synthetic communities consisting of all the identifiable members as a proxy for native communities. Previous studies have also exploited GSMNs from metagenomic data to identify microbial consortia that can perform a specific function with a minimal number of species. Various graph theoretical approaches, including Integer Linear Programming-based solutions for network flow problems^33^, topological sub-network analysis^34^ and network expansion algorithms^35^, used GSMNs to extract the metabolic potential of a community and identify the minimal set of species capable of carrying out a desired function. Most of these methods offer reliable predictions, better scalability and good computational efficiency. However, topological methods provide qualitative solutions without considering the important stoichiometric constraints of reactions in a microbe. To overcome this limitation and gain a better understanding of community behaviour and dynamics through quantitative community features such as microbial growth rate, metabolite production rate and community growth, constraint-based modelling methods are increasingly recognised^26–29,31^.

In this work, we present a systematic mathematical approach to design functionality-dependent minimal microbiomes of a given large community. The minimal microbiome so identified need not be unique—there can be many possible combinations of species that form a minimal microbiome. Knowledge of such minimal microbiomes would be useful in designing treatment strategies for diseases caused by a disruption in the gut microbiome. For example, instead of a faecal transplant to treat *Clostridium difficile* infection, a minimal microbiome with the required functional capability of a large healthy microbiome can be cultivated and administered. This would, in turn, reduce the risk of unintentional transfer of pathogenic microbes^36^. Microbial communities play an important role in bioengineering applications such as chemical production^37^ and waste-water treatment^38,39^, and the concept of the minimal microbiome to recover from perturbations is useful in those situations as well. This work identifies functionality-specific model microbiomes using a constraint-based approach, which could be considered as the ‘community’ analogue of the minimal reactome of an organism^40,41^. It helps to develop customised communities based on a given application. Our algorithm is flexible and allows one to choose the desired functionality, such as the maximisation of the production of a metabolite or the sum of a few metabolites.

## Results and discussion

In this section, we detail the performance of our method, a constraint-based approach to identify minimal microbiomes from a large community. As discussed in the Methods, we use a top-down approach with sequential deletion followed by solving a mixed-integer linear programming (MILP) problem with the objective of minimising the L1-norm of the membership vector, at the same time satisfying certain functionalities that can be defined by the user. We validate and assess the utility of our algorithm by applying it to a synthetic microbial community and three datasets from literature: a gut microbiome dataset, a diet-based minimal gut microbiome dataset and a widely studied synthetic therapeutic consortium. This allows us to comprehensively evaluate the performance of our method and highlight its significance for identifying minimal microbiomes in various contexts.

### Demonstration of the algorithm on a synthetic microbial community

We first evaluate the ‘minMicrobiome’ algorithm on a 9-member community, encompassing both known butyrate producers (*Faecalibacterium prausnitzii*, *Escherichia coli*, *Eubacterium rectale*, and *Bifidobacterium adolescentis*) and non-butyrate producers (*Bartonella quintana, Burkholderiales bacterium, Helicobacter pylori, Clostridium scindens*, and *Blautia wexlerae*). This microbial assembly is predicted to exist as a community with a growth rate of 3.3444 h^−1^ (see Methods) and is capable of producing different SCFAs, including acetate, butyrate, and propionate, at a maximum flux of 0.6694 mmol/gDW-h. The algorithm, under default parameters (gr_opt_frac = 0.99; gr_frac = 0.8; scfa_frac = 0.8; constraint = 1 (sum of all SCFAs); constraint weight = 1:1:1), identifies *E. coli* and *B. wexlerae* as the minimal microbiome for maximal SCFA production of 0.5355 mmol/gDW-h with a community growth of 2.6755 h^−1^. Despite *B. wexlerae* primarily producing acetate rather than butyrate, its inclusion underscores the comprehensive maximisation of all SCFAs. However, while focusing the analysis on butyrate production (Constraint 3), ‘minMicrobiome‘ identifies *E. coli, B. adolescentis*, and *E. rectale* as the minimal microbiome for predominant butyrate production while sustaining community growth. All identified microbes in this analysis are verified butyrate producers capable of supporting community growth. The algorithm also successfully identified the microbe with the highest contribution to butyrate production as a part of a minimal microbiome while reducing redundancy in community functionalities as expected. This focused analysis can be extended to explore the minimal microbiome for various metabolites in diverse microbial ecosystems.

### Analysis of a 9-member community for butyrate production

A complex web of cross-feeding between the microbial species in a community is required to digest the undigested dietary substrates that reach the colon, and to produce SCFA. Despite their intricate exchanges, the gut microbiome presents high functional redundancy and environment-specific variation in inter-species interactions. Butyrate is a crucial SCFA that provides several health benefits to the host due to its anti-carcinogenic^14^ and anti-inflammatory properties^16^. We analyzed a model community with the functionality constraint on maximum butyrate production (Constraint 3) for the gut microbiome consisting of 9 organisms^26^ namely *Bacteroides thetaiotaomicron* VPI 5482 (*Bt*), *Eubacterium rectale* ATCC 33656 (*Er*), *Faecalibacterium prausnitzii* A2 165 (*Fp*), *Enterococcus faecalis* V583 (*Ef*), *Lactobacillus casei* ATCC 334 (*Lc*), *Streptococcus thermophilus* LMG 18311 (*St*), *Bifidobacterium adolescentis* ATCC 15703 (*Ba*), *Escherichia coli* SE11 (*Ec*) and *Klebsiella pneumoniae pneumoniae* MGH78578 (*Kp*), on AGORA high-fibre and Western diets and identified their minimal microbiomes. These organisms were chosen because they can be considered proxies for the four major phyla present in the gut microbiome (Firmicutes, Bacteroidetes, Actinobacteria and Proteobacteria) and are a representative model of the gut microbiome^26^. Analysis of this community to identify minimal microbiomes can shine light on the roles of various species present in the community and can also be validated using known results in literature. The parameter values used are 0.8 for both gr_frac and scfa_frac and 0.99 for gr_opt_frac. The results of the simulation are tabulated in Table 1.

**Table 1:** Predicted minimal microbiomes

Community	Size	High fibre diet		Western diet	
		BPR	*μ*	BPR	*μ*
Gut microbial community	9	10.958	3.646	8.775	3.643
Minimal microbiome	2	8.766	2.917	7.020	2.915
DbMM	10	1.085	1.478	0.980	1.348
Minimal microbiome	3	0.619	1.171	0.793	1.151
Synthetic therapeutic consortium	17	1.588	4.138	1.619	4.149
Minimal microbiome	3	0.734	3.369	0.899	3.413

Table shows three example communities from literature, and predicted minimal microbiomes.

*BPR* Butyrate production rate (mmol/gDW-h); *μ*, growth rate (h^−1^).

On a high-fibre diet, the overall growth rate of this community was calculated to be 3.65 h^−1^, and the individual growth rates were [0.29, 0.007, 0.006, 0.000856, 0.065, 0, 0.27, 2.98, 0.027] h^−1^. The maximum butyrate produced by this community when the growth rate of each species is constrained to be at 99% (gr_opt_frac) of the above individual growth rates was 10.96 mmol/gDW-h. The minimal microbiome that has at least 80% growth (gr_frac) and butyrate production rates (scfa_frac) as that of the above community (Constraint 3) consists of only *Fp* A2 165 and *Ec* SE11. This minimal microbiome can grow at the rate of 2.91 h^−1^ while producing butyrate at 8.77 mmol/gDW-h. Since sub-optimal growth rates increase SCFA production, the constraint of 80% on the growth rate makes the above solution possible. Now, for calculating the maximum butyrate by the community, if 90% of individual growth rates (gr_opt_frac) is used instead of 99%, the butyrate production of the community is 18.79 mmol/gDW-h, with a minimal microbiome comprising *Bt*, *Fp* and *Kp*, that produces 15.03 mmol/gDW-h butyrate. On the other hand, if the lower bound for growth rate (gr_frac) is 90% instead of 80%, the minimal microbiome will have 3.28 h^−1^ growth rate with 8.77 mmol/gDW-h of butyrate production. For this case, there are multiple possible minimal microbiomes containing three organisms: such as *Fp*, *Ba* and *Ec* or *Er*, *Fp* and *Ec*.

**Fig. 1:**
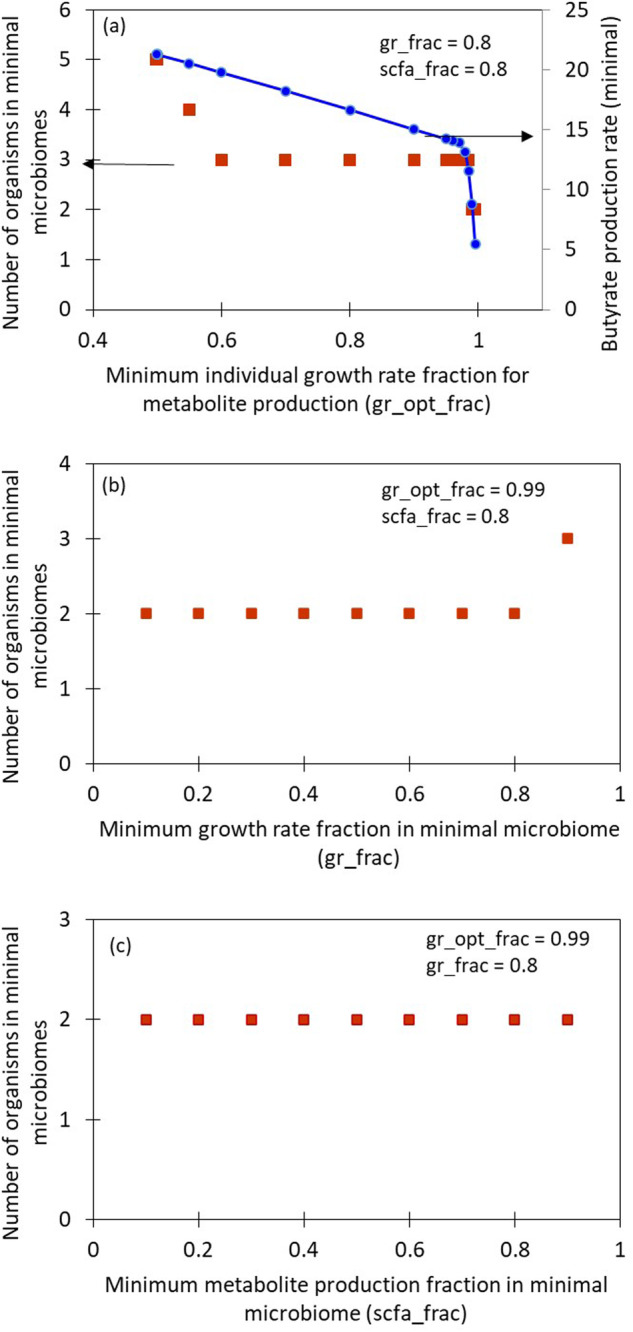
Effect of algorithm parameters on minimal microbiome size and butyrate production rate. The three panels show the number of organisms in the minimal microbiomes when the parameters gr_opt_frac - minimum individual growth rate fraction for metabolite production (**a**), gr_frac - minimum growth rate fraction in minimal microbiome (**b**) and scfa_frac - minimum metabolite production fraction in minimal microbiome (**c**) are varied, for the 9-member community on a high-fibre diet. In (**a**), the corresponding butyrate production rates when gr_opt_frac is varied are indicated on the secondary Y-axis.

The same community (with parameters gr_frac = scfa_frac = 0.8 and gr_opt_frac = 0.99) on a Western diet, has a growth rate of 3.64 h^−1^ with a butyrate production rate of 8.77 mmol/gDW-h. The minimal microbiome is *Fp* and *Ec*, with a growth rate of 2.91 h^−1^. gr_frac and scfa_frac are the fractions of the growth rate and SCFA production rate that are the lower bounds of the growth rate and SCFA production rate in the minimisation problem for finding the minimal microbiomes. Relaxing these parameters could result in more possible combinations of species to form minimal microbiomes. A graphical representation of the effects of the parameters gr_opt_frac, gr_frac and scfa_frac for this 9-member community on high-fiber diet is provided in Fig. 1. A lower value of gr_opt_frac implies that in order to maximise the production of butyrate by the large community, the individual members are allowed to grow at a lower rate. This, in turn, increases the lower bound on the butyrate production rate of the minimal community, and hence more species will be required in the minimal microbiome to achieve the butyrate production requirement. The growth rate of individual species lesser than 0.499 are not plotted because it is not possible to find any minimal microbiomes with such high SCFA production and growth rates (optimisation problem is infeasible). On the Western diet, butyrate production is comparatively lesser, yet the same organisms constitute the minimal microbiome, which is in accordance with the reduction of SCFA in high-fat diets^13^.

It may be noted that all the minimal microbiomes identified consist of either *Fp* or *Er*, which are Firmicutes and are capable of producing butyrate, and are thus the keystone species, in the above community. Belcour et al.^35^ using their function-specific model reduction tool called M2M, have also predicted Firmicutes to be the key organism for butyrate production. Some species, such as the keystone species, could be interchangeable but not essentially equal^21^. The algorithm is not meant to find all the abundant species in the community, but to find a possible minimal community that includes the keystone species for a specific purpose. The keystone species need not be an abundant species in the microbiome.

The constraint on maximising the sum of SCFA (Constraint 1), although intended to find the maximum total SCFA production, sometimes results in the production of only acetate and propionate because the production of acetate is the highest and thus maximises the objective. The code has been enabled for a weighted sum of SCFA where weight can be provided by the user. The aforementioned community of nine organisms was found not to produce acetate and butyrate simultaneously. If the weightage used in the sum of acetate, butyrate and propionate is 1:1:1 or 1:2:1, the production will be 24.554, 0, 0.906 mmol/gDW-h, respectively. On the contrary, if the weightage is 1:3:1, the corresponding fluxes are −1.000, 10.958, 0.906 mmol/gDW-h. If acetate in the diet is removed (by changing the lower bound to 0 from −1), the corresponding fluxes when the weightage is 1:3:1 are 0, 10.732, and 0.910 mmol/gDW-h, respectively. There is only a slight dip in the production of butyrate on the removal of acetate from the diet.

A minimal microbiome does not necessarily identify all the keystone species—so it may be possible to have other microbiomes (not minimal), capable of the same functions. Multiple minimal microbiomes are also possible because multiple combinations of the member species might satisfy the imposed functionality constraints. They could even be mutually exclusive depending on the type of member species. The redundancies in microbial functionalities that result in several possibilities of minimal microbiomes are discussed in detail in ref. ^42^. Thus, the deletion of one of the minimal microbiomes from the large community need not affect the growth rate and functionality of the latter.

### Identification of diet-based minimal gut microbiome

A recent study^43^ identified 10 microbial species as a Diet-based Minimal Microbiome (DbMM) for the effective conversion of dietary fibres to SCFA. The study employed an eco-physiology-guided^44^ approach to identify SCFA-producing (butyrate, propionate) stable minimal microbiomes that could utilise multiple dietary substrates. The identified minimal microbiome embodies functional modules involved in complex carbohydrate degradation to simple sugars and fermentation products and the production of SCFA using the degraded products. Minimal microbiomes and key species under a host-based diet from DbMM are evaluated to illustrate the ability of the proposed algorithm.

The minimal microbiome from 10 core microbial species [*Faecalibaterium prausnitzii*, *Coprococcus catus*, *Bacteroides ovatus*, *Bacteroides xylanisolvens*, *Agathobacter rectalis*, *Anaerobutricum soehngenii*, *Eubacterium siraeum*, *Flavonifactor plautii*, *Roseburia intestinalis*, and *Subdoligranulum variable*] is investigated under different host-based diet conditions (Western and high-fibre) for maximum SCFA (butyrate and propionate) production with optimal community growth. A 10-member microbial community under the Western diet exhibited a growth of 1.35 h^−1^ with 0.921 mmol/gDW-h of propionate production. Butyrate was not predicted to be produced by the community, while optimising for biomass production suggests that the community prefer propionate production over butyrate. Maximum butyrate production of 0.979 mmol/gDW-h was predicted when the growth rate of the community (gr_frac) and individual species (gr_opt_frac) in a community were constrained to 80% and 99%, respectively. A 2-member community of *Bacteroides ovatus* and *Coprococcus catus* is considered a minimal microbiome and they could produce more than 80% of the maximum SCFA produced by the 10-member community. Since the objective is to maximise the linear sum of different SCFA metabolites (propionate and butyrate), the solution obtained showed only propionate production. At a weighted sum of SCFA (propionate: butyrate - 1:1), 0.921 mmol/gDW-h of propionate production is observed with no butyrate production, and the butyrate production of 0.979 mmol/gDW-h at a ratio of 1:5. While optimising for butyrate production, the minimal microbial community includes *B. ovatus* and *C. catus* along with either *Fp* or *Eubacterium* sp. The community captures the cross-feeding of species capable of degrading complex carbohydrates to simple metabolites, such as lactate (*B. ovatus*^45,46^) and a species which could convert the simple metabolites to propionate (*C. catus*^47^) or butyrate (*Fp*^48^, *Er*^49^).

Reducing the constraint for the growth of individual species (gr_opt_frac) in a community to 0.9 instead of 0.99 improves the maximum butyrate production (Constraint 2) to 9.436 mmol/gDW-h ( ≈ 10-fold compared to the results for gr_opt_frac = 0.99), and 4.606 mmol/gDW-h of butyrate production is detected at a ratio of 1:2 (propionate:butyrate). However, the growth of the community drops down to 1.215 h^−1^. Keystone species of the microbial community are consistent, and all three species (*B. ovatus*, *C. catus* and *E. rectale*) are identified as part of the minimal microbiome that facilitates maximum butyrate production.

The same pattern of butyrate and propionate production was observed when the community was simulated on a high-fibre diet. The core microbial community exhibited a growth of 1.479 h^−1^ with 0.901 mmol/gDW-h propionate. The maximum butyrate production of 1.085 mmol/gDW-h is observed at a ratio of 1:5 (propionate: butyrate). *B. ovatus*, an important key microbial species for glycan production, formed a microbial community with *C. catus* and *F. prausnitzii* to produce butyrate and propionate. Few minimal microbiomes also reported the utilisation of *E. rectale* (western diet)/ *Subdoligranulum variabile* (High fibre diet)^50^ as a replacement for butyrate-producing *F. prausnitzii*. A 10-fold increase in butyrate production (10.499 mmol/gDW-h) with decreased growth (1.346 h^−1^) is observed when the individual species’ growth in a community is constrained at 90%. The minimal microbiome analysis suggested that 10 microbial species can produce SCFA from various complex carbohydrates. The proposed algorithm could identify the minimum number of microbial species needed to produce SCFA (desired metabolites) for a specific carbon source.

### Minimal microbiome of a synthetic therapeutic consortium

A synthetic consortium is designed to treat inflammatory bowel disease (IBD) by complementing the missing critical functions of the human gut microbiome. A bottom-up approach-based rational consortium design identified a 17-species therapeutic consortium, including *Megamonas funiformis*, *Megamonas hypermegale*, *Acidaminococcus intestini*, *Bacteroides massiliensis*, *Bacteroides stercoris*, *Barmesiella intestinihominis*, *Fp*, *Subdoligranulum variabile*, *Anaerostipes caccae*, *Anaerostipes hadrus*, *Clostridium symbiosum*, *Akkermansia muciniphila*, *Clostridium scindens*, *Clostridium boltae*, *Blautia producta*, *Blautia hydrogenotropia* and *Marvinbryantia formatexigens*^51^. The consortium is analysed for its therapeutic function to support mucosal homeostasis (SCFA-mediated) and immune modulation defects (bile acid-mediated) in the gut microbiome modulated during the IBD condition. The synthetic community designed to treat IBD and promote gut health has high functional redundancy and complementary auxotrophies for better stability and efficacy. The minimal microbiome of the 17-species consortium for a specific objective will provide insights into the role of microbial species in a community, such as butyrate production, community resilience and secondary bile-acid production.

Here, the algorithm identifies key species involved in the SCFA (propionate and butyrate) mediated energy homeostasis in the microbiome^52^. Default growth parameters are applied to determine the minimum number of microbial species needed to produce the maximum SCFA from the community (gr_opt_frac = 0.99; gr_frac = 0.8; scfa_frac = 0.8). A synthetic consortium exhibits maximum butyrate production of 1.588 mmol/gDW-h and propionate production of 2.382 mmol/gDW-h at a growth rate of 4.138 h^−1^ on a high-fibre diet. Minimal microbiome reveals *C. boltae*, *M. funiformis* and *C. symbiosum* as essential species for SCFA production and can produce nearly 50% of the maximum butyrate (0.734 mmol/gDW-h). The butyrate production increases 13-fold when the growth rate of the community is constrained at 90% instead of 99%. Since the minimal microbiome can vary based on the sequence of the microbes removal from a community, *B. massiliensis*, a propionate producer, replaces the commonly observed propionate producer *M. funiformis* in a few iterations to form a minimal microbiome.

The higher growth rate of the butyrate-producing microbe, *C. symbiosum*^52^ and the propionate-producing microbe, *M. funiformis*^53^, is observed in the minimal microbiome when it is analysed for growth and SCFA production potential. The results suggest that higher butyrate production (1.588 mmol/gDW-h) by the key species (*C. symbiosum*) in 17-member consortia is complemented by the growth of the other microbial species in a community.

A similar trend of butyrate production and growth profile of a community and minimal microbiome on a Western diet is observed (Table 1). The minimal microbiome consists of *C. boltae*, *M. funiformis* and *C. symbiosum*, produces 0.899 mmol/gDW-h of butyrate, at a growth rate of 3.413 h^−1^ and 7.328 mmol/gDW-h at a growth rate of 3.103 h^−1^. The microbiome, including two key species (*M. funiformis* and *C. symbiosum*) accompanied by *C. boltae* to support the growth of individual species and microbiome, is identified as a minimal microbiome for SCFA production irrespective of host diet conditions. Further analysis showed that the other microbial species support the community in fulfilling other significant functions, such as secondary bile acid production and better resilience to pathogen colonisation.

### Minimal microbiomes of a larger model microbiome

We now examine a well-studied model microbiome, to identify its minimal microbiome. Becker et al.^54^ studied simplified human intestinal microbiota (SIHUMI) comprised of *Anaerostipes caccae*, *Bacteroides thetaiotaomicron*, *Bifidobacterium longum*, *Blautia producta*, *Clostridium ramosum*, *Escherichia coli* and *Lactobacillus plantarum*, complemented with *Clostridium butyricum* for butyrate production. They observed improvement in butyrate production with the modified SIHUMIx. The minimal microbiome for this community on a high-fibre diet, with the functionality to maximise butyrate, was found to consist of *Escherichia coli* and *Clostridium butyricum*. The butyrate production by the whole community is 15.16 mmol/gDW-h, whereas that by the minimal microbiome is 12.13 mmol/gDW-h. *Clostridium butyricum* is an irreplaceable member of the minimal microbiome as deletion of this species from the community reduces butyrate production to 11 mmol/gDW-h, which violates the constraint of at least 80% butyrate production.

**Fig. 2:**
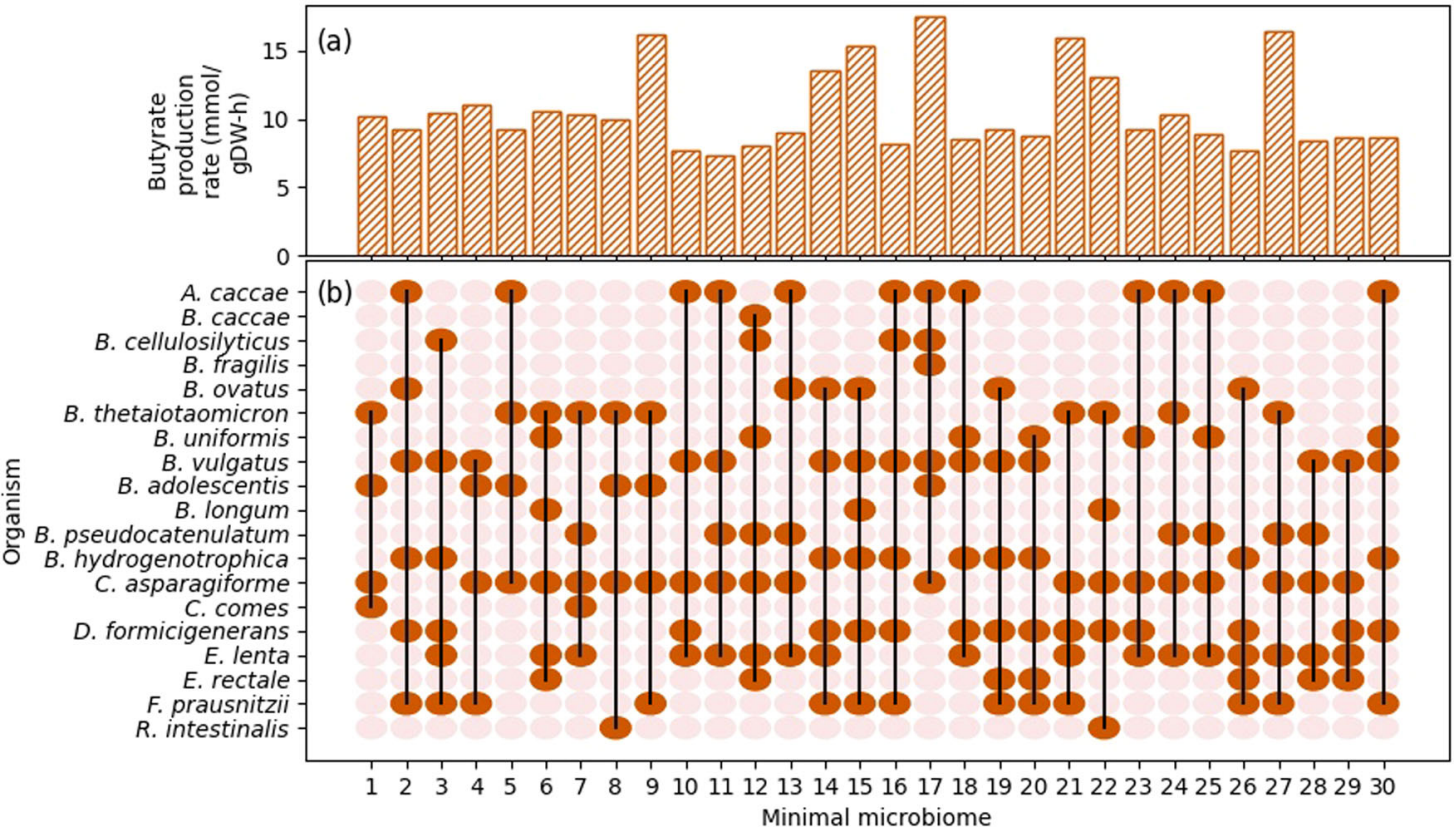
30 minimal microbiomes identified from the 25-member community on the high-fibre diet. The X-axis denotes different minimal microbiomes identified. The presence (dark orange) or absence (light orange) of a species in a minimal microbiome is indicated in the illustration in (**b**), and the corresponding maximum butyrate production rate by each of the minimal microbiomes is shown by the bar graph in (**a**). Only the species present in at least one of the minimal microbiomes are indicated in (**b**).

A community consisting of 25 organisms^55^ with a high-fibre diet was studied, and 30 minimal microbiomes were identified for butyrate production. Figure 2 shows the representation of the presence/absence of all the species and the corresponding butyrate production rates by each minimal microbiome. Some species of *Bacteroides* are capable of breaking down glycans^22^, and thus show up very frequently as a part of the minimal microbiome. Due to the functional redundancy offered by several species, several minimal microbiomes with distinct species are possible and are indicated by the lower frequency appearances. All the 30 minimal microbiomes identified and their maximum butyrate production rates for these conditions are provided in Supplementary Note 1. A table showing a large community of 50 organisms and some of its minimal microbiomes on a high-fibre diet with a constraint on butyrate production is given in Supplementary Note 2.

### Performance of the algorithm

Running the MILP alone for a large community would be computationally intensive. The deletion of one species involves running an LP twice—one for checking the growth rate (FBA) and another (FVA) for the SCFA produced. We observed that when smaller communities were considered, computational times were comparable when solved as an MILP or when size-reduction was done before MILP. For instance, when a 15-member community was run as an MILP (with 15 integer variables and the corresponding continuous flux variables), the time taken for computation was 101 s, whereas when it was reduced to an eight-member community before running MILP, the time taken was 106 s owing to the additional number of LPs. The computations were done on a 2.40 GHz 11th Generation Intel i5-1135G7 processor with 16 GB DDR4 RAM running Windows 11. However, for large communities, the size reduction resulted in significant savings in computational time. MILP of a 25-member community took 1497 s while reducing it to 15 and 8 members reduced the time required to 430 s and 251 s, respectively, on the same machine. It must be noted, however, that the computational time also depends on the complexity of species models.

A known drawback of the algorithm is that it forces the metabolite cross-feeding between the organisms to attain the maximisation objective; nonetheless, it is an admissible assumption in the case of the gut microbiome since it is known to be a co-occurring, cooperative community. If the spatial arrangement of the species or/and the regulatory constraints are known, the individual uptakes for each species can be accordingly adjusted for a more realistic solution. Besides, sub-optimal growth rates are known to result in better SCFA production, and the parameters may be adjusted to suit realistic conditions. In short, the code is flexible to account for a variety of scenarios. The algorithm, however, does not exhaustively identify all the possible minimal microbiomes, especially when multiple functional redundancies are present in the community. While it is readily possible to achieve that by using integer cuts to the MILP problem, there will be a definite drop in performance.

In summary, we presented a procedure of sequential deletion followed by solving an MILP for the identification of a minimal microbiome having specific characteristics of the large microbiome considered. Given a large microbial community, all of its members would not be contributing to certain specific functionalities of the overall microbiome. It is possible to design ‘minimal microbiomes’ specific to certain functionalities, and we present a simple, customisable constraint-based approach for identifying them. Such minimal microbiomes would contain keystone species of the community. Multiple minimal microbiomes, which may even be mutually exclusive, can exist with the capability for a given functionality. Knowledge of minimal microbiomes will be useful in designing microbial composition to treat certain diseases caused by disruption of the gut microbiome. The usage of a rationally designed community for treatment is better than faecal microbial transplantation from a donor that poses a risk for accidental pathogenic infection. The idea of a minimal microbiome can also be made use of in bioengineering applications involving microbial communities, to rescue them from undesired consequences of perturbations. The existing minimal microbiome identification algorithms such as ‘Miscoto’^42^, ‘MultiPus’^34^ and ‘Metage2Metabo’^35^, majorly focus on topological analyses. These algorithms, while effective, do not consider important biological factors such as expected flux through metabolic reactions, interactions between the microbial species and the survival probability of the microbial members in an identified community under a given environment. Being a constraint-based approach, our algorithm can quantitatively predict fluxes and metabolic exchanges and is reasonable for a co-existing community such as the gut microbiome. It presents complementary insights into the structure of microbial communities, as compared to the topological methods, and can be adapted to appropriate objectives, as needed. The algorithm has been designed to be flexible with the possibility of incorporating user-defined inputs for most constraints. The identified minimal microbiomes are found to be rational and include species that digest dietary fibre. Our proposed algorithm is able to identify the minimal microbiomes and keystone species in model communities of the gut and can be readily extended to other scenarios.

## Methods

### Formulation

Genome-scale metabolic models (GSMMs) represent a comprehensive, systems-level metabolic view of an organism by integrating genomic information with biochemical information^56^. Flux Balance Analysis (FBA)^57,58^ is a constraint-based modelling approach that efficiently uses GSMMs to study and explore the cellular metabolism by predicting steady-state flux distributions in an organism under given contexts. Joint FBA^59^ is an extension of FBA for modelling microbial communities by a compartmentalised approach. It solves a linear programming (LP) problem with the defined objective, stoichiometric balances as the equality constraints and lower and upper bounds on fluxes as the inequality constraints. The formulation of joint FBA is as follows:

1 $$\max \mathop{\sum}\limits_{k}{c}^{\top }{v}^{k,biomass}$$

2 $${S}_{ij}^{k}{v}_{j}^{k}=0$$

3 $${LB}_{j}^{k}\le {v}_{j}^{k}\le {UB}_{j}^{k}$$

4 $${{v}_{j}}^{exchange}=\mathop{\sum}\limits_{k}({v}_{j}^{k,exchange})$$

where *k* denotes species, *j* denotes reactions, *i* denotes metabolites, *S* is the stoichiometric matrix, and *v* are the reaction fluxes. In this approach, the overall exchange of each metabolite by the community is the sum of the corresponding exchange reactions of each organism, and these constraints enable metabolite uptakes and cross-feeding. However, in this approach, if the objective is to maximise the sum of biomass reaction fluxes of each organism, it could result in metabolite production without growth for certain organisms, which is unrealistic. This problem is surmounted by coupling the metabolic reactions of an organism to its biomass equation:

5 $${v}_{j}^{k}-c\cdot {v}^{k,biomass}\le u\quad \forall k\in species$$

where *c* is the coupling vector and *u* is the threshold.

Due to the mathematical formulation to maximise the sum of biomass, constraint-based approaches such as joint FBA might force metabolite cross-feeding to attain the maximum possible growth. For co-occurring species in the gut microbiome, this cross-feeding would be reasonable, unless the species are known to be regulated differently or spatially separated.

To find the minimal microbiome, we define a binary membership vector identifying the status of membership of each species as 0: absent, or 1: present. This integer vector is used in the biomass constraint. The problem is now an MILP and is solved with the objective of minimising the *L*_1_-norm of the membership vector with the required functionality constraints. The following approach is used to find the functionality constraints:

1. The overall and individual growth rates of the given large community are calculated by solving the joint FBA LP problem.
2. The maximum possible rates of production of the desired metabolites—by default, SCFA (acetate, butyrate and propionate), or their sum, is calculated by Flux Variability Analysis (FVA) by keeping the individual growth rates calculated in the previous step as the lower bounds for the individual biomass fluxes. The code is designed to work with the production of any three individual metabolites or their weighted sum as the *constraint*. The objective function for FVA is:
  6 $$\max ({w}_{ac}{v}_{ac}^{exchange}+{w}_{bu}{v}_{bu}^{exchange}+{w}_{pr}{v}_{pr}^{exchange})$$
  where *w* denotes the weightage for each of the fluxes. The default weightage is (1, 1, 1) and can be varied by the user if the production of one product is preferred to the others. The additional constraint on individual growth rates is given by:
  7 $${v}^{k,biomass}\ge gr\,{{{\_}}}opt{{{\_}}}\,frac\times constant$$
  where the constant value is obtained from the joint FBA solution of Step 1. If needed, a different metabolite list can be provided by the user in the optional inputs. The fraction of growth rates to be considered as lower bounds in this step can also be provided by the user (default value of ‘gr_opt_frac’ is 0.99). This value is relevant because a sub-optimal growth rate is known to be more realistic and results in good SCFA production^60^. In the code, the constraint on the sum of SCFA is denoted as Constraint 1. Likewise, constraints on the production of acetate, butyrate and propionate are denoted as Constraints 2, 3 and 4, respectively.
3. Fractions of individual growth rate calculated in Step 1 and SCFA production rate calculated in Step 2 are provided as the lower bounds for the corresponding fluxes in the MILP problem. The growth rate fraction (gr_frac) and SCFA production fraction (scfa_frac) can be provided by the user.

The MILP formulation is as follows:

8 $$\min \mathop{\sum}\limits_{k}{X}^{k}$$

9 $${S}_{ij}^{k}{v}_{j}^{k}=0$$

10 $${LB}^{k,biomass}{X}^{k}\le {v}^{k,biomass}\le {UB}^{k,biomass}{X}^{k}$$

11 $${LB}_{j}^{k}\le {v}_{j}^{k}\le {UB}_{j}^{k};j\ne biomass$$

12 $${v}_{j}^{k}-c.{v}^{k,biomass}\le u$$

13 $${v}_{j}^{k}\in \left(-\infty ,\infty \right)$$

14 $${X}^{k}\in \left(0,1\right\}$$

Functionality and growth rate constraints are captured as follows:

15 $${v}_{SCFA}^{exchange}\ge scf{a}_{frac}.{\vartheta }_{SCFA}^{exchange}$$

16 $${v}^{k,biomass}\ge g{r}_{frac}.{\vartheta }^{k,biomass}$$

where *k* denotes species, *j* denotes reactions, *i* denotes metabolites, *S* is the stoichiometric matrix, *X* are the integer (binary membership) variables and *v* are the reaction fluxes (continuous variables). *LB* and *UB* denote the lower and upper bounds. *ϑ* in functionality constraints denotes reaction fluxes of the full community.

For large communities, solving an MILP is computationally challenging. In such cases, organisms can be deleted one by one in a given sequence such that the functionality constraints are satisfied by the resultant community, until the community is small enough for reasonable computation for MILP solution. If the deletion sequence is not provided by the user, a random sequence is chosen by default. The deletion sequence is run over multiple times to reach the MILP size provided by the user. ‘Deletion’ of an organism is done by assigning zero values to the lower and upper bounds of all the corresponding fluxes of the organism. However, the resultant minimal microbiome will depend on the sequence used for deletion and hence multiple iterations of the algorithm help to identify different possible solutions.The effect of deletion sequence in identifying the minimal microbiomes is demonstrated in Supplementary Note 3. The number of organisms in the minimal microbiomes identified in different iterations may vary. If required by the user, the code also has a feature to compute the maximum value of the SCFA production rate by the minimal communities.

**Fig. 3:**
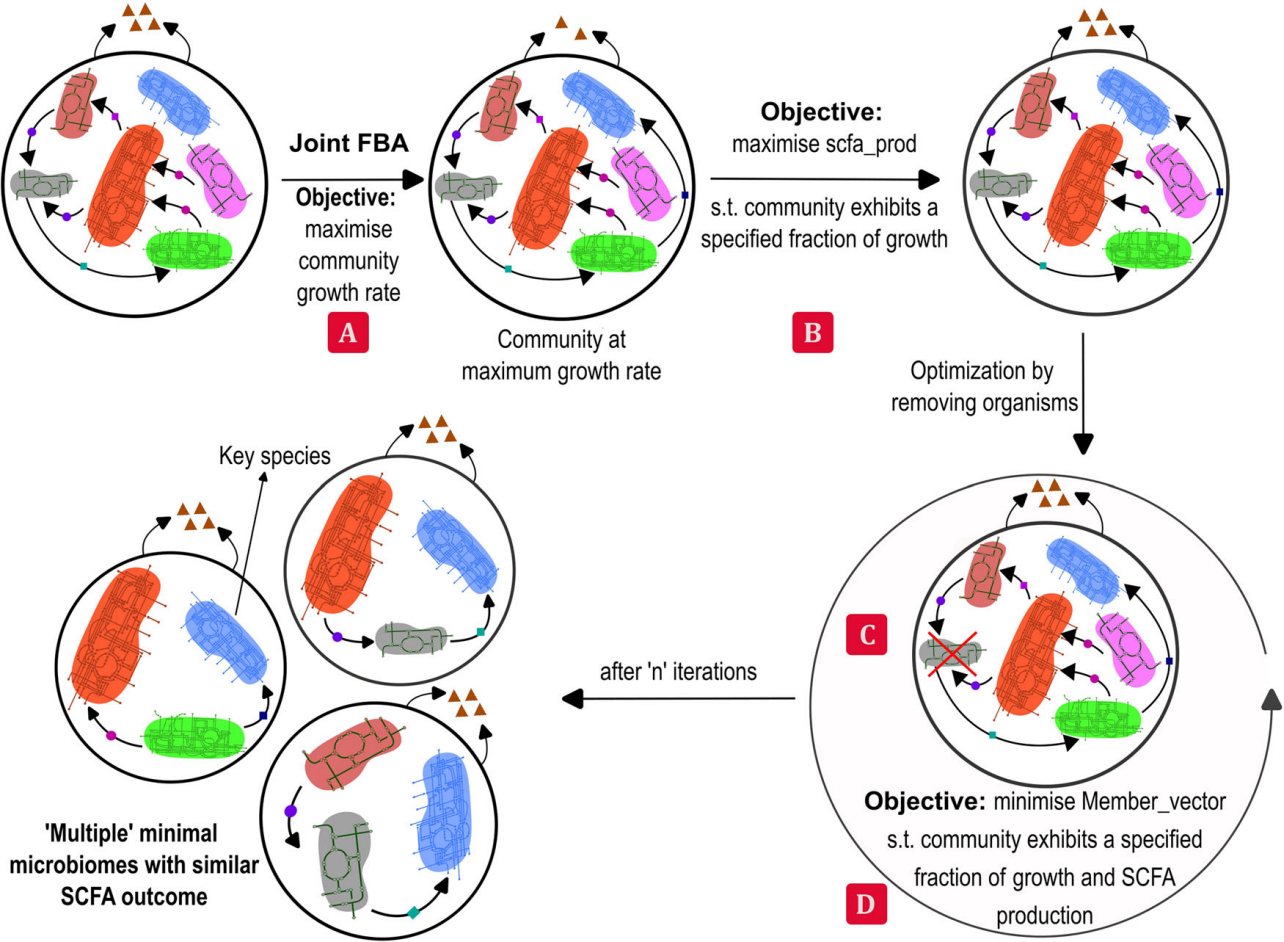
Schematic illustration of the proposed algorithm. Every circle above illustrates a microbiome with different microbes, each having its own metabolic network. At each step, indicated by the coloured squares, various steps described in the formulation are illustrated. Triangles indicate secreted metabolites from each of the microbiomes (objective, such as SCFA production). The minimisation is done in steps **C** and **D**, employing constraints based on the growth and production rates obtained from steps **A** and **B**.

The algorithm is explained in step-by-step detail in Supplementary Methods, and a schematic is provided in Fig. 3. COBRA toolbox^61^ functions are used in MATLAB 2020b for the computations. The exchange and biomass reactions are identified in the code by the names used in AGORA^62^ models, and each individual organism in the community is identified as ‘_org<number>’ as in the communities created using the COBRA function createCommModel(). Gurobi (version 9.1.2, Gurobi Optimisation LLC, USA) is the solver used for solving both LP and MILP.

### Generation of synthetic community to validate ‘minMicrobiome’ algorithm

We employed a synthetic community comprising nine microorganisms to evaluate the ‘minMicrobiome’ algorithm, which identifies the minimal set of microorganisms within a community capable of producing a desired metabolic function. The microorganisms within this synthetic community were deliberately selected to encompass key butyrate and acetate producers, as well as those unable to produce acetate and butyrate. Using community FBA in MATLAB, we validated the growth of all microorganisms within the community and analysed the production of desired metabolites like SCFAs. The microorganisms are selected based on their capability for acetate and butyrate production: (i) Acetate and butyrate producers -*Escherichia coli, Bifidobacterium adolescentis, Faecalibacterium prausnitzii*, and *Eubacterium rectale*; (ii) Butyrate producers - *Helicobacterpylori*; (iii) Acetate producers -*Blautia wexlerae* and *Clostridium scindens*; and (iv) butyrate and acetate non-producers - *Bartonella quintana*and *Burkholderiales bacterium*. The algorithm aims to identify the key contributors to acetate/butyrate production, supporting the growth of a minimal set of organisms within a community. Although seven microbes with potential for inclusion in the minimal microbiome were added, the algorithm is anticipated to select organisms with higher metabolite production rates, minimising functional redundancy within the community.

### Reporting summary

Further information on research design is available in the Nature Research Reporting Summary linked to this article.

### Supplementary information

Supplementary Material

Reporting summary

## Data Availability

All data generated or analysed during the study is provided in the Supplementary Material.

## Code availability

The ‘minMicrobiome’ algorithm is available from https://github.com/RamanLab/minMicrobiome.
