# Supplementary material for: Designing function-specific minimal microbiomes from large microbial communities
Source: NPJ Syst Biol Appl. 2024 May 3;10:46. doi: 10.1038/s41540-024-00373-1 (PMC11068740; doi:10.1038/s41540-024-00373-1)
Supplement: Supplementary file 1 — Supplementary Material [file 41540_2024_373_MOESM1_ESM.pdf]

Designing function-specific minimal microbiomes from large microbial communities

Aswathy K. Raghu[1,2], Indumathi Palanikumar[1,2,3], Karthik Raman[1,2,3,4*]

[1]Centre for Integrative Biology and Systems mEdicine (IBSE), Indian Institute of Technology (IIT) Madras, Chennai — 600 036, India

[2]Robert Bosch Centre for Data Science and Artificial Intelligence (RBCDSAI), IIT Madras, Chennai 600 036, India

[3]Department of Biotechnology, Bhupat Jyoti Mehta School of Biosciences, IIT Madras, Chennai 600 036, India

[4]Wadhwani School of Data Science at AI, IIT Madras, Chennai 600 036.

∗To whom correspondence should be addressed.

Supplementary information:

- **Supplementary Methods**: A detailed explanation of the `minMicrobiome' algorithm
- **Supplementary Note 1**: Minimal microbiomes of different communities
- **Supplementary Note 2:** Minimal microbiome for a large community
- **Supplementary Note 3:** Demonstration of the effect of deletion sequence

Supplementary Methods: A detailed explanation of the `minMicrobiome' algorithm

Inputs:

1. Community model of AGORA models created using `createCommModel` :–
   - Each species should be represented as '`_org1`', '`_org2`', etc.
   - Exchange reactions should start with '`EX_`'
   - Biomass equation names should start with '`biomass`'

2. Provide the constraints on exchanges (intake and generation) in lb (lower bounds) and ub (upper bounds).

Optional inputs: In a structure '`options`'

1. Fraction of growth rate to be retained in minimal microbiome – **`gr_frac` (default value = 0.8)**
2. Fraction of SCFA to be retained in minimal microbiome – **`scfa_frac` (default value = 0.8)**
3. For coupling biomass reaction to the others in a species: coupling constraint, **`c` (default value = 1000)** and threshold **`u` (default value = 0.01)**
4. The functionality of the community that needs to be preserved – **`constraint`**: -

    `1`: maximum weighted sum of SCFAs (metabolites 1, 2 and 3, default '**`wt`**' = 1:1:1),

    `2`: max metabolite 1 production

    `3`: maximum metabolite 2 production

    `4`: maximum metabolite 3 production

    (**default '`constraint`' value = 1. Default '`met_names`': metabolite 1 = acetate, metabolite 2 = butyrate and metabolite 3 = propionate**)
5. Maximum number of organisms for MILP – **`maxMILP` (default value = 10)**
6. Total no. of iterations the code should run (the no. minimal microbiomes to be identified) – **`iter` (default value = 3)**
7. Number of times the MILP should be run with different initial conditions – **`MILP_runs` (default value = 1; keep this value to be <= number of organisms)**
8. Maximum number of rounds the generated random sequence should be run over for

deletion (MILP may not reduce to the desired number with 1 round) – **`max_del_rounds` (default value = 10)**

9. Fraction of original growth rates for constraint while finding maximum SCFA production of the original community - **`gr_opt_frac` (default value = 0.99)**
10. Sequence for deletion - **`del_seq` (generated randomly by default)**
11. Does the metabolite production rate need to be calculated – yes or no? - **`met_calc_minMicrobiome` (default value = 'yes')**
12. What is the maximum time up to which you would like it to run (seconds) – **`time_budget` (default value = 10800 s)**

Output:

- **`solutionPert`** – a cell containing the identified minimal microbiomes (No. of minimal microbiomes calculated <= `iter * ` **`MILP_runs`**)
- **`supp_out`** – additional output containing the list of all distinct minimal microbiomes identified, maximum production rate by each of them if applicable, the full community growth rate (`gr_max`), maximum individual growth rates and maximum SCFA production (`max_scfa`) of the original community, list of organisms removed during the initial deletion and the deletion sequence

Algorithm:

1. Add coupling constraints to each species present in the community – couple each biomass reaction to the other reactions in the species
2. Joint FBA solution (`optimizeCbModel`) of the community
3. Identify '`scfa_list`', which is the array of indices of metabolites considered for the constraint on functionality.

4. Find the maximum SCFA (`max_scfa`) possible from the community by Flux Variability Analysis (based on the constraint):

   Add the following constraint to a copy of the community model:

   individual growth rates of organisms >= `gr_opt_frac*growth rates from the FBA solution`

   Make the objective function as maximization of the reactions in 'scfa_list' with weightage ('wt') also under consideration.

   Solve the LP using solveCobraLP. The value of fluxes through the exchange reactions in the **'scfa_list'** is the '`max_scfa`'.

5. If `max_scfa < tol` (1E-3), display "The community does not produce metabolite 'x'" and return. Else, proceed to the next step.

6. Deletions to reduce the number of organisms to <= `maxMILP`

   a. If `del_seq` is not given, generate a random sequence of numbers from 1 to total no. of organisms in the community

   b. Remove the organism corresponding to the first no. in `del_seq` by changing the reaction bounds of all reactions of that organism to 0.

   c. Perform joint FBA.

   d. If the growth rate of the new community is >= `gr_frac`* growth rate of the original community, add the constraints on biomass and perform SCFA maximization LP as in step 4.

      If `scfa_new` satisfies the constraint on SCFA (if it is >= `scfa_frac`* value of original community), remove that organism. Else keep it and continue with steps 6.b – 6.d for the next organism in `del_seq`.

e. Repeat until the number of organisms reaches `maxMILP` or the number of deletion cycles reaches `max_del_rounds`.

7. If no. of organisms > '`milp_max`', display "MILP > '`milp_max`'!" and go to the next iteration. '`milp_max`' is a parameter defined in minMicrobiome to restrict the MILP size for reasonable computation times. Default value is 50 and can be edited by the user inside the function.

else

a. Add binary variables (`X`)– one for each organism originally present.

Add the following constraint to each organism:

b. If the organism is deleted after steps 5 and 6, the corresponding `X` is constrained to 0

c. Add constraint on SCFA - >= **`scfa_frac`** * value of scfa from original community

d. Add constraint on overall growth – `gr_frac`*community max growth

e. Change the objective function to minimize the sum of `X`s.

f. For different initial guess values for the solution, solve MILP using `solveCobraMILP`. Run this loop for "`MILP_runs`" number of times

8. If the identified solution is not redundant, add it to `solutionPert`. If not, continue with the next iteration.

9. If `met_calc_minMicrobiome` is yes,

a. Start with the original large community.

b. If the binary variables corresponding to an organism in the community is 0, set all the corresponding reactions of that organism in the community to 0.

c. Set constraint on overall growth – `gr_frac`*community max growth

d. Find maximum SCFA as in step 4.

Supplementary Note 1: Minimal microbiomes of different communities

Minimal microbiome from 9-member communities:

Community	Minimal microbiome
Gut microbial community (9 organisms, HFD and WD)	
Bacteroides thetaiotaomicron VPI 5482	*Faecalibacterium prausnitzii A2 165*
Eubacterium rectale ATCC 33656	*Escherichia coli SE11*
Faecalibacterium prausnitzii A2 165	
Enterococcus faecalis V583	
Lactobacillus casei ATCC 334	
Streptococcus thermophilus LMG 18311	
Bifidobacterium adolescentis ATCC 15703	
Escherichia coli SE11	
Klebsiella pneumoniae pneumoniae MGH78578	
**SIHUMIx**	
Anaerostipes caccae	*Escherichia coli*
Bacteroides thetaiotaomicron	*Clostridium butyricum*
Bifidobacterium longum	
Blautia producta	
Clostridium ramosum	
Escherichia coli	
Lactobacillus plantarum	
Clostridium butyricum	

Community of 25 species

25 species in the large community:

**1**	*Prevotella copri CB7 DSM 18205*
**2**	*Parabacteroides johnsonii DSM 18315*
**3**	*Bacteroides vulgatus ATCC 8482*
**4**	*Bacteroides fragilis 3 1 12*
**5**	*Bacteroides ovatus ATCC 8483*
**6**	*Bacteroides thetaiotaomicron VPI 5482*
**7**	*Bacteroides caccae ATCC 43185*
**8**	*Bacteroides cellulosilyticus DSM 14838*
**9**	*Bacteroides uniformis ATCC 8492*
**10**	*Desulfovibrio piger ATCC 29098*
**11**	*Bifidobacterium longum infantis ATCC 15697*
**12**	*Bifidobacterium adolescentis ATCC 15703*
**13**	*Bifidobacterium pseudocatenulatum DSM 20438*
**14**	*Collinsella aerofaciens ATCC 25986*
**15**	*Eggerthella lenta DSM 2243*

**16**	*Faecalibacterium prausnitzii A2 165*
**17**	*Clostridium hiranonis TO 931 DSM 13275*
**18**	*Anaerostipes caccae DSM 14662*
**19**	*Blautia hydrogenotrophica DSM 10507*
**20**	*Clostridium asparagiforme DSM 15981*
**21**	*Eubacterium rectale ATCC 33656*
**22**	*Roseburia intestinalis L1 82*
**23**	*Coprococcus comes ATCC 27758*
**24**	*Dorea longicatena DSM 13814*
**25**	*Dorea formicigenerans ATCC 27755*

Community features on high fiber diet:-

Community growth rate	4.498 h^{-1}
Community butyrate rate	9.806 mmol/gDW-h
minMicrobiome growth rate	3.599 h^{-1}
minMicrobiome butyrate rate	7.845 mmol/gDW-h

The minimal microbiomes identified for this large community and the maximum butyrate production rate by each minimal microbiome is given in the following table. The butyrate production rate is calculated based on Flux Variability Analysis.

**Sr. no.**	**Minimal Microbiomes**	**Butyrate Rate (mmol/gDW-h)**
1	*Bacteroides thetaiotaomicron VPI 5482*	10.1758
	Bifidobacterium adolescentis ATCC 15703	
	Clostridium asparagiforme DSM 15981	
	Coprococcus comes ATCC 27758	
2	*Bacteroides vulgatus ATCC 8482*	9.1849
	Bacteroides ovatus ATCC 8483	
	Faecalibacterium prausnitzii A2 165	
	Anaerostipes caccae DSM 14662	
	Blautia hydrogenotrophica DSM 10507	
	Dorea formicigenerans ATCC 27755	
3	*Bacteroides vulgatus ATCC 8482*	10.3972
	Bacteroides cellulosilyticus DSM 14838	
	Eggerthella lenta DSM 2243	
	Faecalibacterium prausnitzii A2 165	
	Blautia hydrogenotrophica DSM 10507	
	Dorea formicigenerans ATCC 27755	

4	*Bacteroides vulgatus ATCC 8482*	11.0209
	Bifidobacterium adolescentis ATCC 15703	
	Faecalibacterium prausnitzii A2 165	
	Clostridium asparagiforme DSM 15981	
5	*Bacteroides thetaiotaomicron VPI 5482*	9.1732
	Bifidobacterium adolescentis ATCC 15703	
	Anaerostipes caccae DSM 14662	
	Clostridium asparagiforme DSM 15981	
6	*Bacteroides thetaiotaomicron VPI 5482*	10.5071
	Bacteroides uniformis ATCC 8492	
	Bifidobacterium longum infantis ATCC 15697	
	Eggerthella lenta DSM 2243	
	Clostridium asparagiforme DSM 15981	
	Eubacterium rectale ATCC 33656	
7	*Bacteroides thetaiotaomicron VPI 5482*	10.2777
	Bifidobacterium pseudocatenulatum DSM 20438	
	Eggerthella lenta DSM 2243	
	Clostridium asparagiforme DSM 15981	
	Coprococcus comes ATCC 27758	
8	*Bacteroides thetaiotaomicron VPI 5482*	9.9939
	Bifidobacterium adolescentis ATCC 15703	
	Clostridium asparagiforme DSM 15981	
	Roseburia intestinalis L1 82	
9	*Bacteroides thetaiotaomicron VPI 5482*	16.1896
	Bifidobacterium adolescentis ATCC 15703	
	Faecalibacterium prausnitzii A2 165	
	Clostridium asparagiforme DSM 15981	
10	*Bacteroides vulgatus ATCC 8482*	7.7235
	Eggerthella lenta DSM 2243	
	Anaerostipes caccae DSM 14662	
	Clostridium asparagiforme DSM 15981	
	Dorea formicigenerans ATCC 27755	
11	*Bacteroides vulgatus ATCC 8482*	7.3016

	Bifidobacterium pseudocatenulatum DSM 20438	
	Eggerthella lenta DSM 2243	
	Anaerostipes caccae DSM 14662	
	Clostridium asparagiforme DSM 15981	
12	*Bacteroides caccae ATCC 43185*	8.0201
	Bacteroides cellulosilyticus DSM 14838	
	Bacteroides uniformis ATCC 8492	
	Bifidobacterium pseudocatenulatum DSM 20438	
	Eggerthella lenta DSM 2243	
	Clostridium asparagiforme DSM 15981	
	Eubacterium rectale ATCC 33656	
13	*Bacteroides ovatus ATCC 8483*	8.9832
	Bifidobacterium pseudocatenulatum DSM 20438	
	Eggerthella lenta DSM 2243	
	Anaerostipes caccae DSM 14662	
	Clostridium asparagiforme DSM 15981	
14	*Bacteroides vulgatus ATCC 8482*	13.4692
	Bacteroides ovatus ATCC 8483	
	Eggerthella lenta DSM 2243	
	Faecalibacterium prausnitzii A2 165	
	Blautia hydrogenotrophica DSM 10507	
	Dorea formicigenerans ATCC 27755	
15	*Bacteroides vulgatus ATCC 8482*	15.3684
	Bacteroides ovatus ATCC 8483	
	Bifidobacterium longum infantis ATCC 15697	
	Faecalibacterium prausnitzii A2 165	
	Blautia hydrogenotrophica DSM 10507	
	Dorea formicigenerans ATCC 27755	
16	*Bacteroides vulgatus ATCC 8482*	8.1928
	Bacteroides cellulosilyticus DSM 14838	
	Faecalibacterium prausnitzii A2 165	
	Anaerostipes caccae DSM 14662	
	Blautia hydrogenotrophica DSM 10507	
	Dorea formicigenerans ATCC 27755	

17	*Bacteroides vulgatus ATCC 8482*	17.4334
	Bacteroides fragilis 3 1 12	
	Bacteroides cellulosilyticus DSM 14838	
	Bifidobacterium adolescentis ATCC 15703	
	Anaerostipes caccae DSM 14662	
	Clostridium asparagiforme DSM 15981	
18	*Bacteroides vulgatus ATCC 8482*	8.5122
	Bacteroides uniformis ATCC 8492	
	Eggerthella lenta DSM 2243	
	Anaerostipes caccae DSM 14662	
	Blautia hydrogenotrophica DSM 10507	
	Dorea formicigenerans ATCC 27755	
19	*Bacteroides vulgatus ATCC 8482*	9.2782
	Bacteroides ovatus ATCC 8483	
	Faecalibacterium prausnitzii A2 165	
	Blautia hydrogenotrophica DSM 10507	
	Eubacterium rectale ATCC 33656	
	Dorea formicigenerans ATCC 27755	
20	*Bacteroides vulgatus ATCC 8482*	8.7895
	Bacteroides uniformis ATCC 8492	
	Faecalibacterium prausnitzii A2 165	
	Blautia hydrogenotrophica DSM 10507	
	Eubacterium rectale ATCC 33656	
	Dorea formicigenerans ATCC 27755	
21	*Bacteroides thetaiotaomicron VPI 5482*	15.922
	Eggerthella lenta DSM 2243	
	Faecalibacterium prausnitzii A2 165	
	Clostridium asparagiforme DSM 15981	
	Dorea formicigenerans ATCC 27755	
22	*Bacteroides thetaiotaomicron VPI 5482*	13.0593
	Bifidobacterium longum infantis ATCC 15697	
	Clostridium asparagiforme DSM 15981	
	Roseburia intestinalis L1 82	
	Dorea formicigenerans ATCC 27755	
23	*Bacteroides uniformis ATCC 8492*	9.2794

	Eggerthella lenta DSM 2243	
	Anaerostipes caccae DSM 14662	
	Clostridium asparagiforme DSM 15981	
	Dorea formicigenerans ATCC 27755	
24	*Bacteroides thetaiotaomicron VPI 5482*	10.2578
	Bifidobacterium pseudocatenulatum DSM 20438	
	Eggerthella lenta DSM 2243	
	Anaerostipes caccae DSM 14662	
	Clostridium asparagiforme DSM 15981	
25	*Bacteroides uniformis ATCC 8492*	8.8215
	Bifidobacterium pseudocatenulatum DSM 20438	
	Eggerthella lenta DSM 2243	
	Anaerostipes caccae DSM 14662	
	Clostridium asparagiforme DSM 15981	
26	*Bacteroides ovatus ATCC 8483*	7.6738
	Eggerthella lenta DSM 2243	
	Faecalibacterium prausnitzii A2 165	
	Blautia hydrogenotrophica DSM 10507	
	Eubacterium rectale ATCC 33656	
	Dorea formicigenerans ATCC 27755	
27	*Bacteroides thetaiotaomicron VPI 5482*	16.4438
	Bifidobacterium pseudocatenulatum DSM 20438	
	Eggerthella lenta DSM 2243	
	Faecalibacterium prausnitzii A2 165	
	Clostridium asparagiforme DSM 15981	
28	*Bacteroides vulgatus ATCC 8482*	8.3977
	Bifidobacterium pseudocatenulatum DSM 20438	
	Eggerthella lenta DSM 2243	
	Clostridium asparagiforme DSM 15981	
	Eubacterium rectale ATCC 33656	
29	*Bacteroides vulgatus ATCC 8482*	8.5918
	Eggerthella lenta DSM 2243	
	Clostridium asparagiforme DSM 15981	

	Eubacterium rectale ATCC 33656	
	Dorea formicigenerans ATCC 27755	
30	*Bacteroides vulgatus ATCC 8482*	8.6648
	Bacteroides uniformis ATCC 8492	
	Faecalibacterium prausnitzii A2 165	
	Anaerostipes caccae DSM 14662	
	Blautia hydrogenotrophica DSM 10507	
	Dorea formicigenerans ATCC 27755	

Supplementary Note 2: Minimal microbiome for a large community

In the following tables, a community consisting of 50 members and its minimal microbiomes on high fiber diet with a constraint on maximizing butyrate production are shown. The growth rate of the full community is 4.92 h^{-1} with butyrate production of 19.7 mmol/gDW-h and that of the minimal microbiome is 3.94 h^{-1} with butyrate production of 15.83 mmol/gDW-h.

1	*Akkermansia muciniphila ATCC BAA 835*
2	*Anaerostipes caccae DSM 14662*
3	*Bacteroides vulgatus ATCC 8482*
4	*Bacteroides fragilis 3 1 12*
5	*Bacteroides ovatus ATCC 8483*
6	*Bacteroides thetaiotaomicron VPI 5482*
7	*Bacteroides caccae ATCC 43185*
8	*Bacteroides cellulosilyticus DSM 14838*
9	*Bacteroides uniformis ATCC 8492*
10	*Bifidobacterium longum infantis ATCC 15697*
11	*Bifidobacterium adolescentis ATCC 15703*
12	*Bifidobacterium pseudocatenulatum DSM 20438*
13	*Blautia hydrogenotrophica DSM 10507*
14	*Blautia producta DSM 2950*
15	*Butyrivibrio fibrisolvens 16 4*
16	*Clostridium hiranonis TO 931 DSM 13275*
17	*Clostridium asparagiforme DSM 15981*
18	*Clostridium clostridioforme CM201*
19	*Clostridium innocuum 2959*
20	*Clostridium spiroforme DSM 1552*
21	*Clostridium scindens ATCC 35704*
22	*Clostridium ramosum VPI 0427 DSM 1402*
23	*Clostridium butyricum DSM 10702*
24	*Collinsella aerofaciens ATCC 25986*
25	*Coprococcus comes ATCC 27758*
26	*Desulfovibrio piger ATCC 29098*
27	*Dorea longicatena DSM 13814*
28	*Dorea formicigenerans ATCC 27755*
29	*Eggerthella lenta DSM 2243*
30	*Enterococcus faecalis V583*
31	*Escherichia coli SE11*
32	*Eubacterium rectale ATCC 33656*

33	*Faecalibacterium prausnitzii A2 165*
34	*Flavonifractor plautii ATCC 29863*
35	*Klebsiella pneumoniae pneumoniae MGH78578*
36	*Lactobacillus acidophilus ATCC 4796*
37	*Lactobacillus casei ATCC 334*
38	*Lactobacillus salivarius HO66 ATCC 11741*
39	*Lactobacillus plantarum JDM1*
40	*Lactobacillus reuteri CF48 3A*
41	*Oxalobacter formigenes HOxBLS*
42	*Parabacteroides distasonis ATCC 8503*
43	*Parabacteroides johnsonii DSM 18315*
44	*Prevotella copri CB7 DSM 18205*
45	*Pseudoflavonifractor capillosus strain ATCC29799*
46	*Roseburia intestinalis L1 82*
47	*Ruminococcus torques ATCC 27756*
48	*Ruminococcus obeum A2 162*
49	*Streptococcus thermophilus LMG 18311*
50	*Staphylococcus xylosus DMB3 Bh1*

Community growth rate	4.925 h^{-1}
Community butyrate rate	19.7907 mmol/gDW-h
minMicrobiome growth rate	3.9401 h^{-1}
minMicrobiome butyrate rate	15.8326 mmol/gDW-h

**Sr. no.**	**Minimal Microbiomes**	**Butyrate Rate (mmol/gDW-h)**
1	*Bacteroides thetaiotaomicron VPI 5482*	17.097
	Clostridium innocuum 2959	
	Flavonifractor plautii ATCC 29863	
	Klebsiella pneumoniae pneumoniae MGH78578	
2	*Bacteroides uniformis ATCC 8492*	16.9509
	Clostridium clostridioforme CM201	
	Clostridium butyricum DSM 10702	
	Escherichia coli SE11	
3	*Bacteroides cellulosilyticus DSM 14838*	18.6642
	Clostridium clostridioforme CM201	
	Clostridium butyricum DSM 10702	
	Escherichia coli SE11	
4	*Bacteroides cellulosilyticus DSM 14838*	18.7575

	Clostridium clostridioforme CM201	
	Clostridium innocuum 2959	
	Escherichia coli SE11	
5	*Bacteroides ovatus ATCC 8483*	17.4256
	Dorea formicigenerans ATCC 27755	
	Escherichia coli SE11	
	Faecalibacterium prausnitzii A2 165	
6	*Bacteroides thetaiotaomicron VPI 5482*	17.9326
	Clostridium innocuum 2959	
	Dorea formicigenerans ATCC 27755	
	Escherichia coli SE11	
7	*Bacteroides ovatus ATCC 8483*	19.0953
	Clostridium innocuum 2959	
	Escherichia coli SE11	
	Flavonifractor plautii ATCC 29863	
8	*Bacteroides vulgatus ATCC 8482*	19.7826
	Bacteroides uniformis ATCC 8492	
	Clostridium clostridioforme CM201	
	Clostridium innocuum 2959	
	Klebsiella pneumoniae pneumoniae MGH78578	
9	*Bacteroides vulgatus ATCC 8482*	17.7504
	Bacteroides thetaiotaomicron VPI 5482	
	Flavonifractor plautii ATCC 29863	
	Klebsiella pneumoniae pneumoniae MGH78578	
10	*Bacteroides vulgatus ATCC 8482*	15.6484
	Bacteroides caccae ATCC 43185	
	Flavonifractor plautii ATCC 29863	
	Klebsiella pneumoniae pneumoniae MGH78578	

Supplementary Note 3: Demonstration of the effect of deletion sequence

Solving the entire problem as an MILP using the branch and bound method will point to the minimal microbiome, but sequential deletions in the beginning helps to identify the numerous possible options of the solutions. The sequence of deletion affects the result, and this feature can be leveraged to identify various possible minimal microbiomes.

We are demonstrating this for the case of the 9-member gut microbial community on high-fiber diet using `gr_frac` = 0.7 (not the default value) and `scfa_frac` = 0.8.

The list of species in the large community

1	*Bacteroides thetaiotaomicron VPI 5482*
2	*Eubacterium rectale ATCC 33656*
3	*Faecalibacterium prausnitzii A2 165*
4	*Enterococcus faecalis V583*
5	*Lactobacillus casei ATCC 334*
6	*Streptococcus thermophilus LMG 18311*
7	*Bifidobacterium adolescentis ATCC 15703*
8	*Escherichia coli SE11*
9	*Klebsiella pneumoniae pneumoniae MGH78578*

The community and minimal microbiome growth rates and butyrate production rates are as follows.

Community growth rate	3.646 h^{-1}
Community butyrate rate	10.958 mmol/gDW-h
minMicrobiome growth rate	2.5526 h^{-1}
minMicrobiome butyrate rate	8.7665 mmol/gDW-h

When the model of 9 organisms is run as an MILP with an initial guess as all integer variables = 1, the minimal microbiome consists of the following species.

Eubacterium rectale ATCC 33656
Klebsiella pneumoniae pneumoniae MGH78578

In a different approach, one member was deleted first and the remaining model of 8 members was solved as an MILP with an initial guess as all integer variables = 1. The identified minimal microbiomes when each member was deleted first are shown below.

Deletion	Resultant Minimal Microbiome
1	*Eubacterium rectale ATCC 33656*
	Escherichia coli SE11
2	*Faecalibacterium prausnitzii A2 165*
	Klebsiella pneumoniae pneumoniae MGH78578
3	Cannot be removed, so it works like a regular 9 member MILP
4	*Eubacterium rectale ATCC 33656*
	Escherichia coli SE11
5	*Eubacterium rectale ATCC 33656*
	Escherichia coli SE11
6	*Eubacterium rectale ATCC 33656*
	Klebsiella pneumoniae pneumoniae MGH78578
7	*Eubacterium rectale ATCC 33656*
	Escherichia coli SE11
8	Cannot be removed, so it works like a regular 9 member MILP
9	*Faecalibacterium prausnitzii A2 165*
	Escherichia coli SE11

It may be noticed that the deletion sequence affects the identified minimal microbiome and. This is why a random sequence of deletion is used as it eliminates possible bias on deletion sequence when multiple runs are done to identify different microbiomes. It also proves the effectiveness of our methodology in identifying multiple minimal microbiomes. If the user would like to choose a deletion sequence, we would recommend sticking to one where the least important ones are deleted first, so that the algorithm could lead them to a minimal microbiome.

If any species in the deletion sequence is so important that the growth rate and butyrate rate constraints cannot be met, the code avoids that deletion and informs the user.

The reason why *Fp* does not always appear in the minimal microbiomes even though the code doesn't allow its deletion is because deletion is based on stricter constraints than that present in MILP. That is, individual growth rate constraints are enforced to calculate the growth rate and butyrate production rate to be considered for deletion, whereas overall growth rate constraint is only applied in the MILP step. This is done to ensure that the important species are not deleted in the deletion step and that we arrive at the smallest minimal microbiome in the MILP step.
